# Supplementary material for: Therapeutic adenine base editing of human hematopoietic stem cells
Source: Nat Commun. 2023 Jan 13;14:207. doi: 10.1038/s41467-022-35508-7 (PMC9839747; doi:10.1038/s41467-022-35508-7)

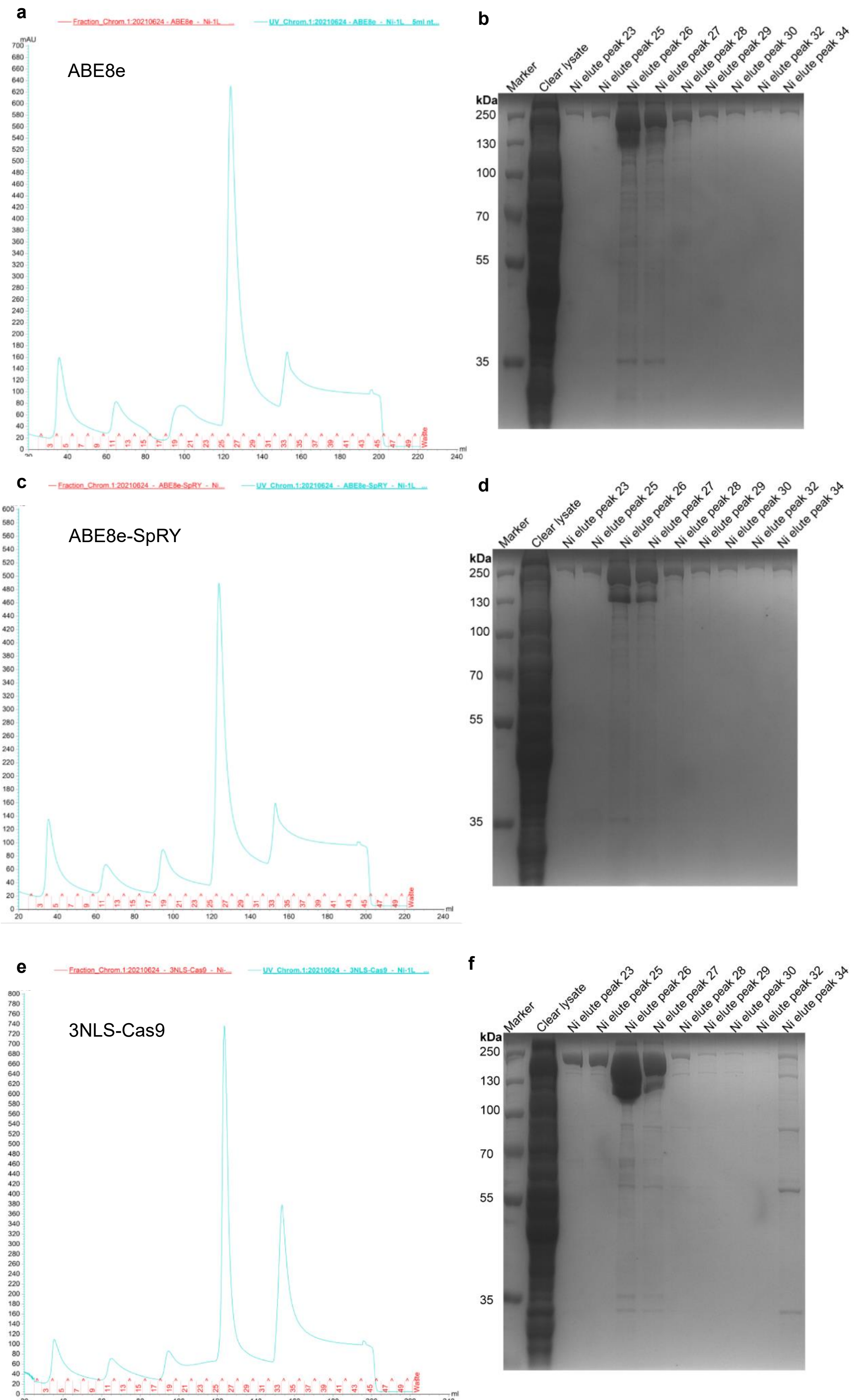
a
ABE8e
mAU
ml
b
Marker
Clear lysate
Ni elute peak 23
Ni elute peak 25
Ni elute peak 26
Ni elute peak 27
Ni elute peak 28
Ni elute peak 29
Ni elute peak 30
Ni elute peak 32
Ni elute peak 34
kDa
250
130
100
70
55
35
c
ABE8e-SpRY
d
e
3NLS-Cas9
f

g

h

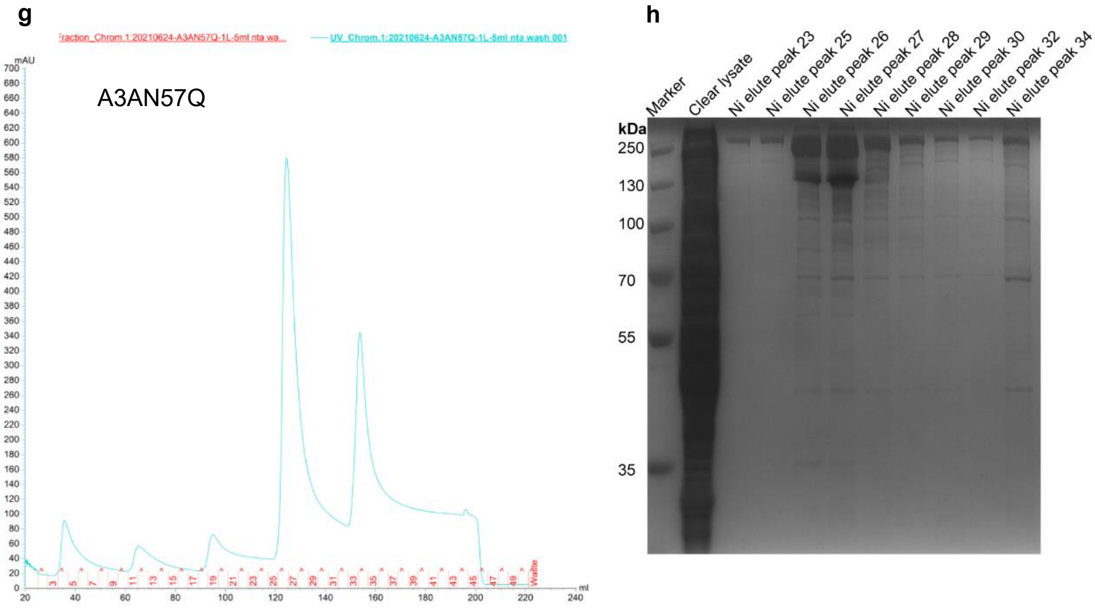

Supplementary Fig. 1 | Purification of ABE8e, ABE8e-SpRY, 3NLS-Cas9, A3AN57Q. **a**, **c**, **e**, **g**, Purification strategy and profiles. Proteins was purified using nickel affinity. Desired fractions 26-27 from imidazole elution (nickel column tube 26–27) were collected and then dialysis were performed. **b**, **d**, **f**, **h**, Protein purity validation. Protein purity was determined using SDS-PAGE and gel staining. n=3 experiments were repeated independently with similar results.

a

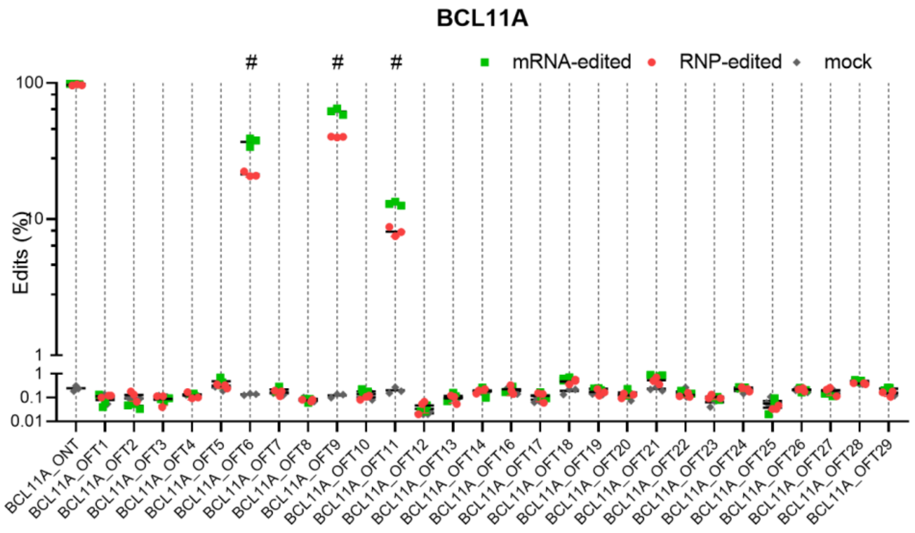

b

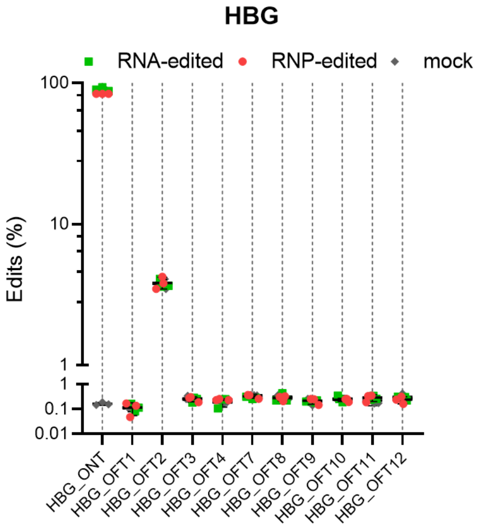

Supplementary Fig. 2 | Guide RNA-dependent off-target potential of ABE8e editing targeting sg1620 and sgHBGsense, respectively. a, Using the CasOFFinder tool, 28 potential genomic off-target sites with 3 or fewer mismatches to the on-target BCL11A enhancer sequence were identified. Each site was evaluated by amplicon deep sequencing. Dot graphs show the percentage of sequencing reads containing A•T-to-G•C mutations within protospacer positions 1–11 at on- and off-target sites in genomic DNA samples from patient CD34^{+} HSPCs treated with ABE8e mRNA, protein, or untreated controls (n = 3). # indicates off-target sites with difference in edit frequency between mock and edited samples of at least 0.1% (effective off-target). **b,** Using the Cas-OFFinder tool, 10 potential genomic off-target sites with 3 or fewer mismatches to the on-target HBG1/2 promoter sequence were identified. Each site was evaluated by amplicon deep sequencing. Each input sample is from an independent experiment.

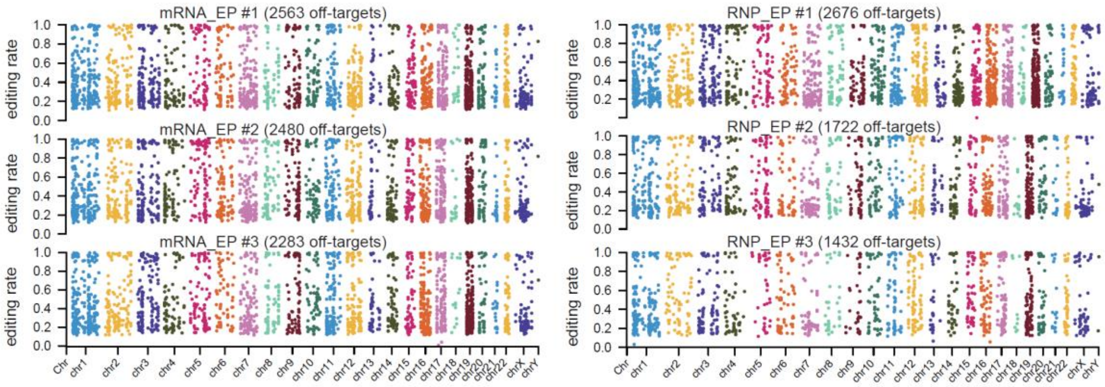

Supplementary Fig. 3 | Comparison of the off-target RNA SNVs for mRNA and RNP groups. Distributions of off-target RNA SNVs on human chromosomes for mRNA and RNP. Chromosomes are indicated with different colours. EP, electroporation. mRNA, ABE8e mRNA +sg1620. RNP, ABE8e protein+sg1620. n=3 biological independent samples.

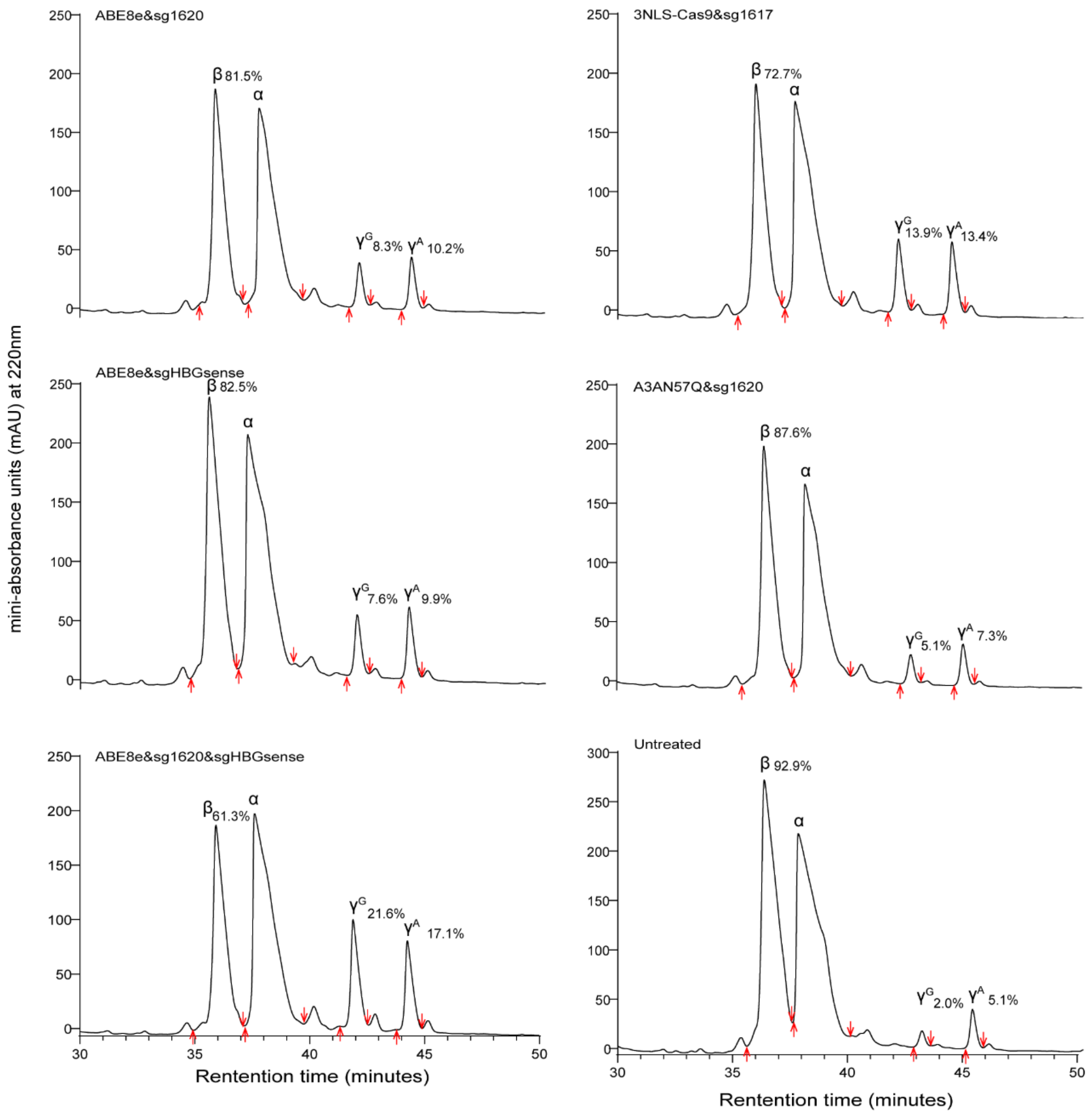

Supplementary Fig. 4 | Reverse-phase HPLC analysis of erythroid cells derived from in vitro differentiation of edited CD34⁺ HSPCs. Reverse-phase HPLC chromatograms of erythroid cell lysates at culture day 18, with β-like globins and their associated fractions marked near the associated peak. Protein levels of β-like globins by genome editing mediated by ABE8e-sg1620, ABE8e-sgHBGsense, ABE8e-sg1620&sgHBGsense, A3A (N57Q)-BE3–sg1620 and 3×NLS-SpCas9–sg1617 targeting the same core half E-box/GATA motif at +58 BCL11A erythroid enhancer were analyzed. Four independent experiments were performed. Red arrows indicate the start and end of globin chain peaks.

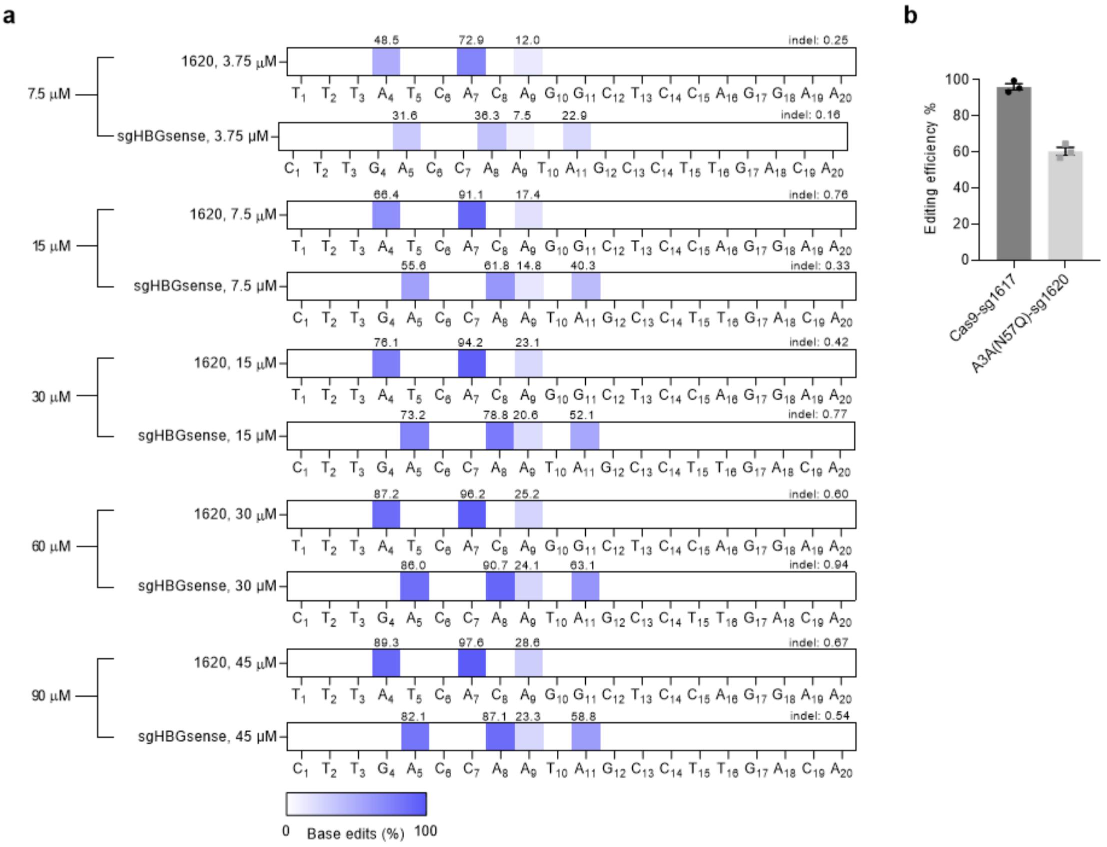

Supplementary Fig. 5 | Editing efficiency of ABE8e, A3AN57Q and 3NLS-Cas9. a, Dose-dependent A base editing by ABE8e–sg1620&sgHBGsense RNP electroporation of HSPCs. Base edits quantified by deep sequence analysis. **b,**Editing efficiency of A3AN57Q in complex with sg1620 and indels created by 3NLS-Cas9-sg1617. Frequencies of editing outcomes were quantified using CRISPResso2 software and collapsed on the basis of mutations in the quantification window. Indels overlapping the spacer sequence were counted as indels, and C>N substitutions at spacer positions 1–10 were counted as base edits for total edit quantification. For base editor, indels were excluded before calculation of nucleotide substitution frequency. Data were presented as mean ± sd, n=3 independent experiments.

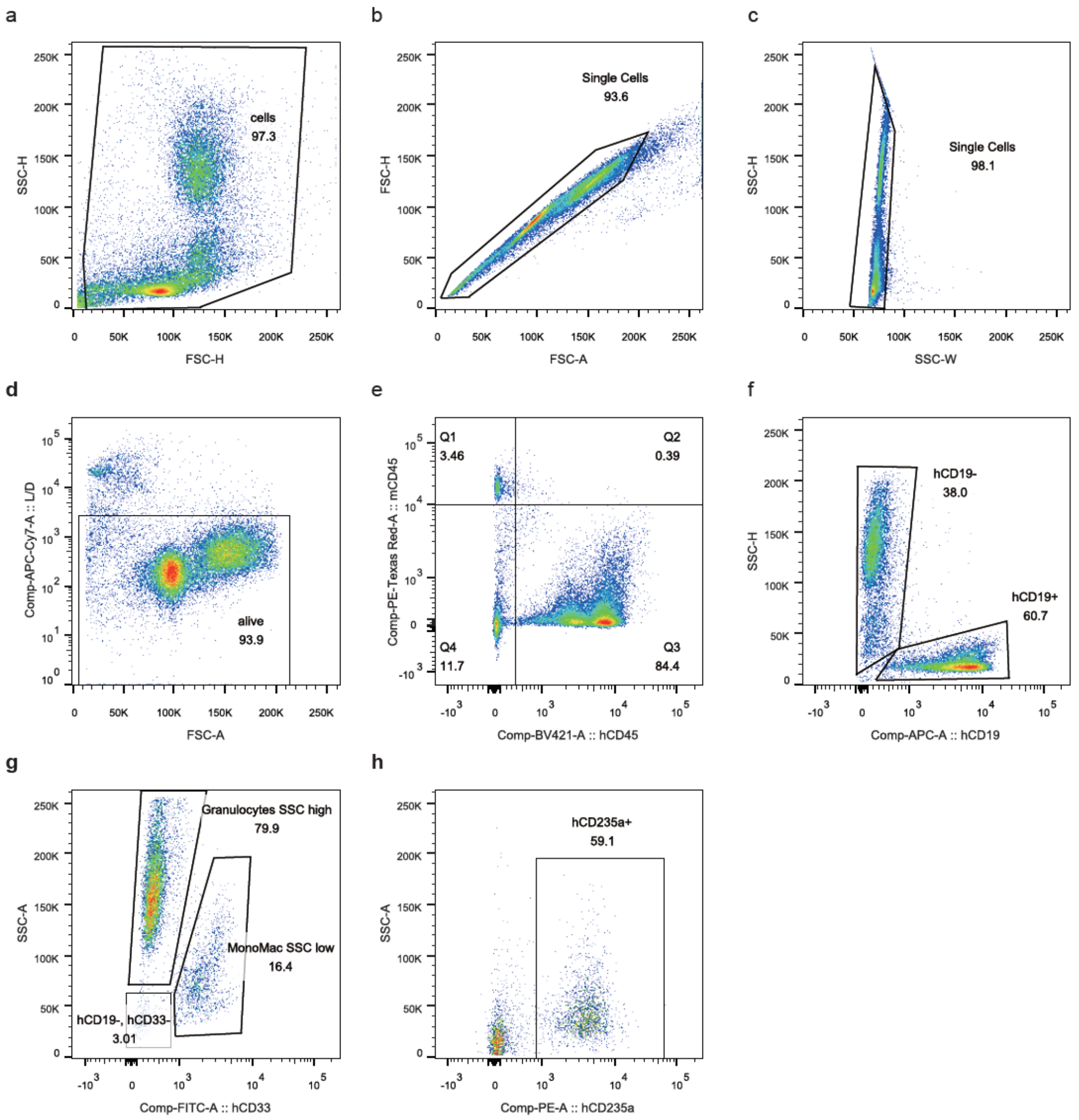

Supplementary Fig. 6 | Representative xenografted bone marrow flow cytometry analysis. a–d, Live cells from engrafted mouse BM. **e**, Human cells gated from hCD45$^+$ population, mouse cells gated from mCD45$^+$ population. **f**, B cells gated from hCD45$^+$CD19$^+$ population. **g**, Granulocytes gated from hCD45$^+$ CD19$^-$CD33dim with SSC high population. Monocytes gated from hCD45$^+$CD19$^-$CD33$^+$ with SSC low population. **h**, Erythroid cells gated from hCD45$^-$mCD45$^-$ hCD235a$^+$ population.

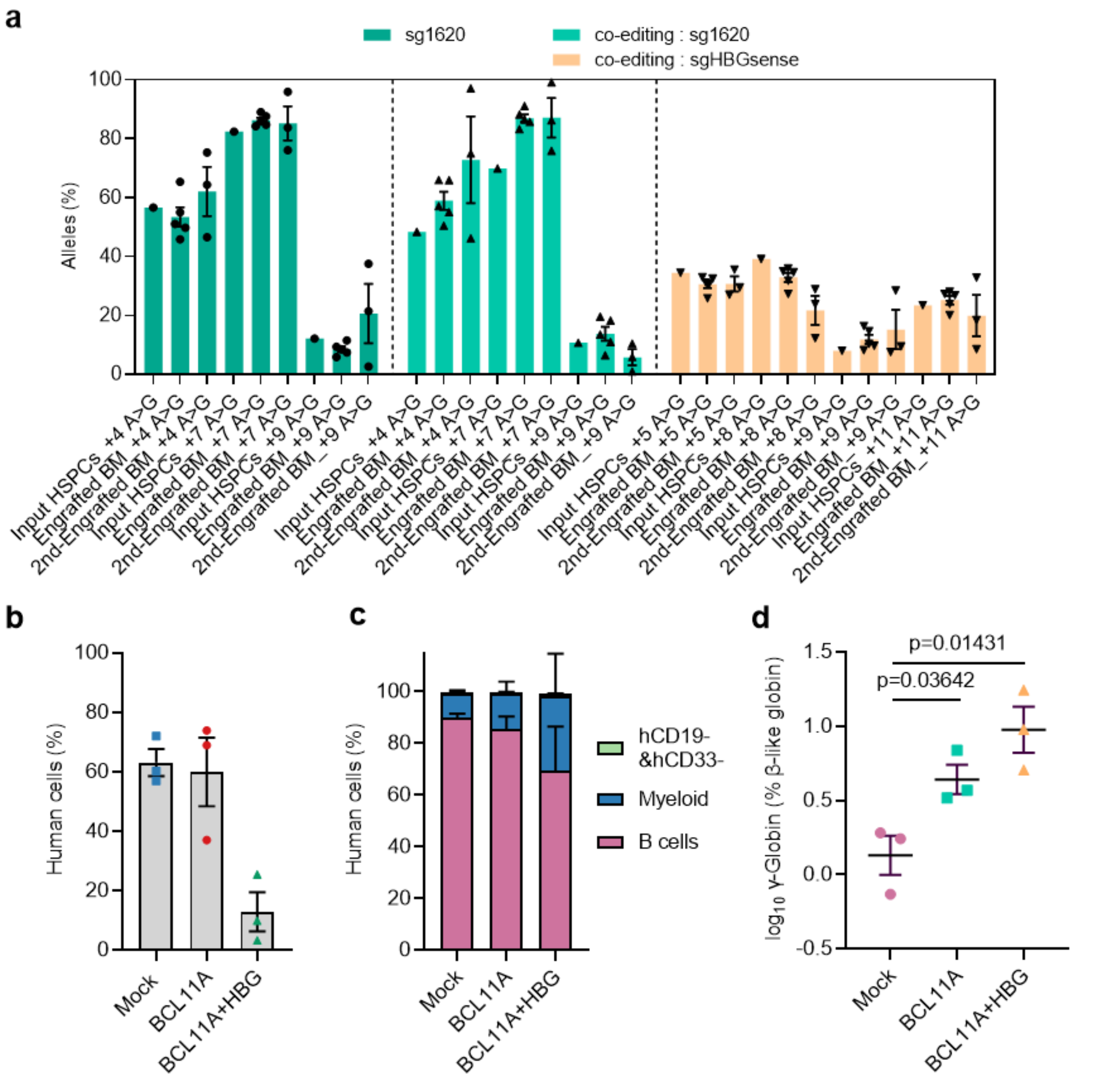

Supplementary Fig. 7 | Efficient and durable A>G base editing in HSCs from healthy donor. **a**, Base editing in unfractionated BM from mice 16 weeks after secondary transplantation as compared to input HSPCs and the first engrafted BM cells. Data are plotted as mean ± sd. n = 5 primary recipients for each group of engrafted HSPCs, and n=3 for secondary recipients. **b**, After extraction of bone marrow cells from the primary transplanted mice, each group of cells was mixed and then divided into three equal parts for secondary transplantation. Human BM chimerism were analyzed 16 weeks after base edited HSPC infusion. Data are plotted as mean ± sd, n=3 mice from mock and all edited groups. **c**, Percentage of secondary engrafted human B cells, myeloid cells and $CD19^{-}CD33^{-}$ cells 16 weeks after transplantation. Data were presented as mean ± sd, n=3 mice for mock or edited group. **d**, γ-globin induction analyzed by RT–qPCR normalized by β-like globin, measured from secondary transplantation BM chimerism 16 weeks after infusion. Data are log transformed and plotted as mean ± sd, analyzed with the unpaired two-tailed Student's t-test, p-values have been noted on the corresponding comparisons. n = 3 replicates from individual recipient mice.

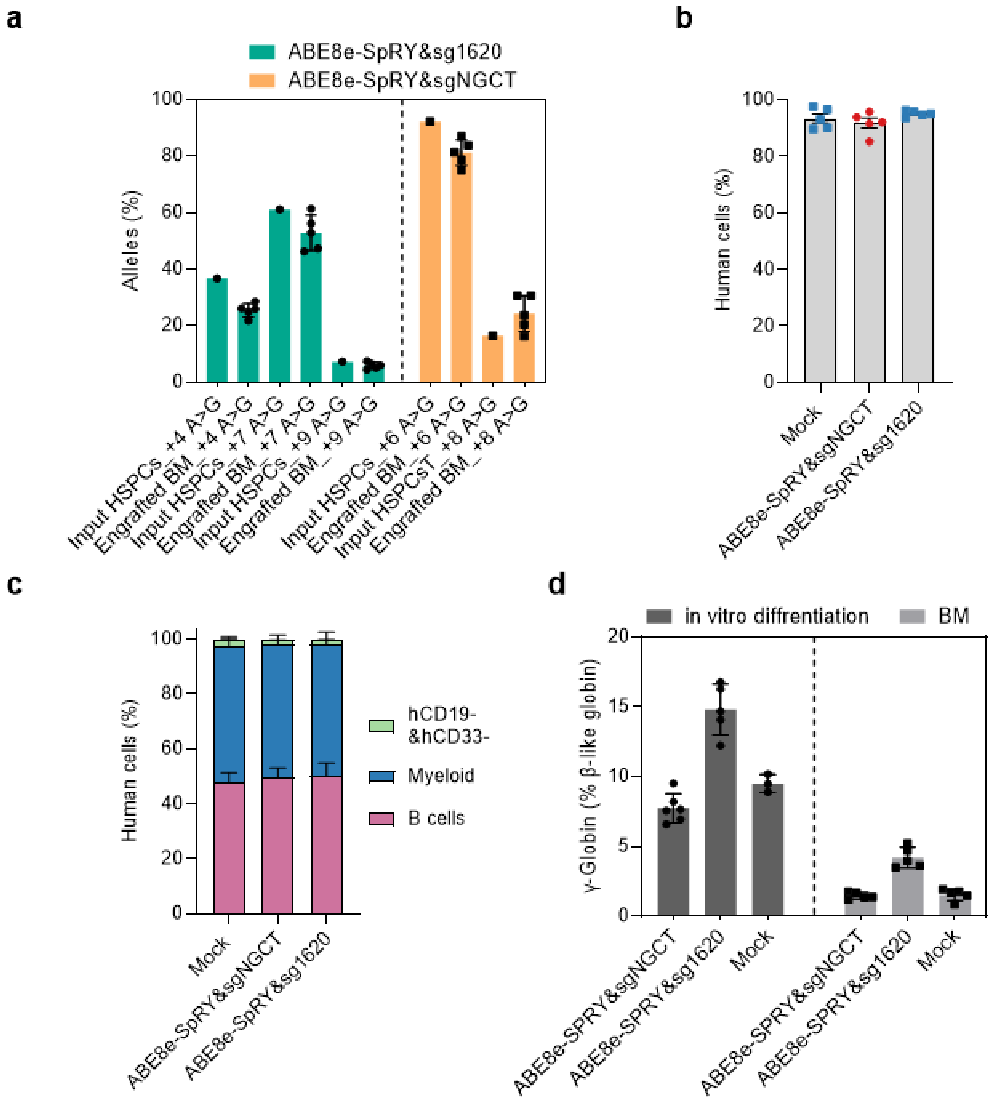

Supplementary Fig. 8 | ABE8e-SpRY editing performance at sg1620 and sgNGCT testing loci. **a**, Base editing of ABE8e-SpRY at 1620 or NGCT site in unfractionated BM after 16 weeks as compared to input HSPCs. Data are plotted as mean ± sd. n = 5 mice for engraftment. **b**, Human BM chimerism were analyzed 16 weeks after infusion, measured from grafts of HSPCs from health donor edited by ABE8e-SpRY targeted to 1620 and NGCT sites, respectively. Data are plotted as mean ± sd, n = 5 mice for mock and each edited group. **c**, Percentage of engrafted human B cells, myeloid cells and $CD19^{-}CD33^{-}$ cells, measured from grafts of HSPCs edited by ABE8e-SpRY targeted to 1620 or NGCT site. Data are plotted as mean ± sd. n = 5 mice for mock and each edited group. **d**, RT-qPCR of ABE8e-SpRY induced γ-globin gene expression relative to β-like globin expression of erythroid progeny after in vitro differentiation and bone marrow transplantation. For the in vitro differentiation, three to six independent experiments were performed in technical triplicate, and data are shown as mean ± sd. For the bone marrow transplantation, 5 primary recipients of each group were analyzed in technical triplicate with similar results.

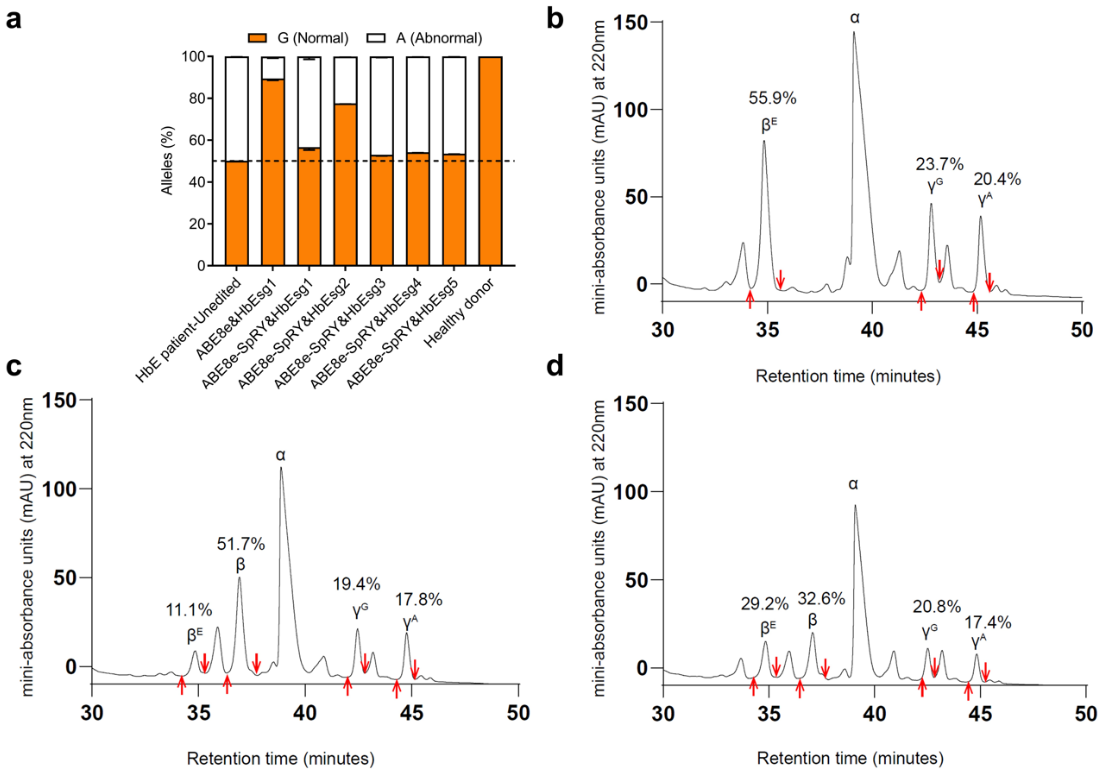

Supplementary Fig. 9 | ABE8e-SpRY therapeutic base editing in β-thalassaemia/HbE patient CD34+ HSPCs. **a**, Allele frequency of target sites after ABE8e or ABE8e-SpRY editing in $CD34^+$ HSPCs from Hb E/β-thalassaemia patients. Data are plotted as mean ± sd. n=3 replicates from independent experiments. **b-d**, Reverse-phase HPLC chromatograms of erythroid cell lysates at in vitro diffrentiation culture day 18, with β-like globins and their associated fractions marked near the associated peak. The independent experiment was repeated 3 times (HbE #1-3), and representative results are shown (HbE #3). Red arrows indicate the start and end of globin chain peaks. **b**, HbE patient-unedited; **c**, ABE8e&HbEsg1; **d**, ABE8e-SpRY&HbEsg2.

a

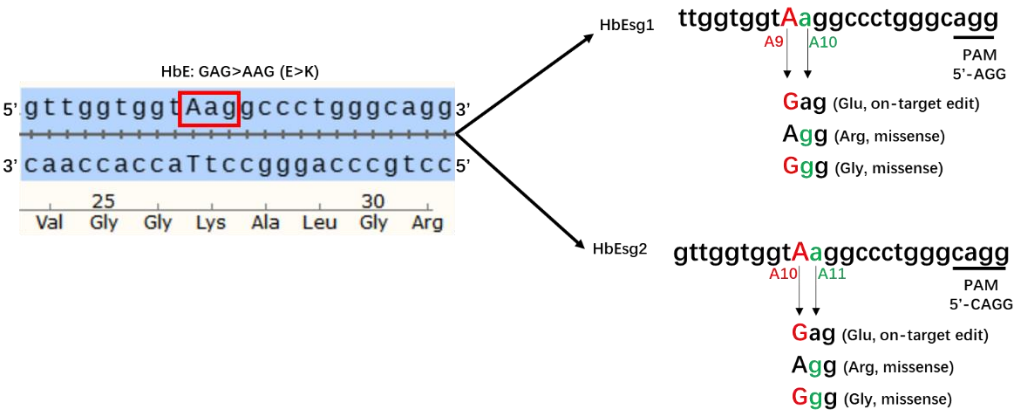

b

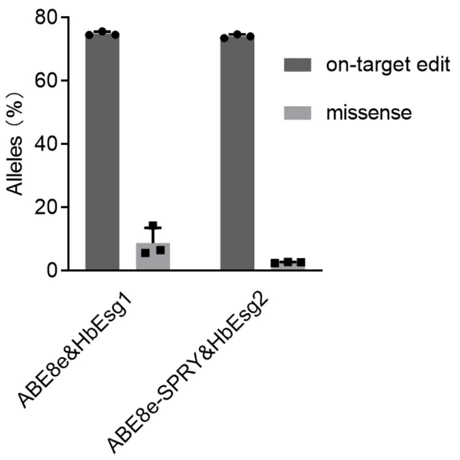

Supplementary Fig. 10 | Adenine base editing converts HbE β-globin gene to wild-type β-globin gene in patient CD34⁺ HSPCs. CD34⁺ cells from patients with HbE variant were electroporated with ABE8e RNP using HbEsg1 and ABE8e-SpRY RNP using HbEsg2, respectively, targeting the HbE mutant HBB codon. **a,** The edited region of HBB with the target A shown in red along with potential bystander A edits in green. **b,** Editing efficiencies determined by deep sequencing at target and bystander adenines after 4d in stem-cell culture medium after electroporation (HbE #1-3). Data were presented as mean ± sd, n=3 independent experiments.

Supplementary Table. 1 Summary of donor cell genotypes used in this study

Donor cell	donor gender	genotype	β type	Context nucleotide sequence
β-thalassaemia HSPCs (β0β+ #1)	male	$\beta^{CD41/42(-CTTT)}$	β^0	TGGTCTACCCTTGGACCCAGAGGTT**[-CTTT]**GAGTCCTTTGGGGATCTGTCC
		β^{-28}	β^+	GGAGGGCAGGAGCCAGGGCTGGGCATA**G**AAGTCAGGGCAGAGCCATCTATT
HbE #1 HSPCs	male	β^{CD17}	β^0	GTCTGCCGTTACTGCCCTGTGGGGC**T**AGGTGAACGTGGATGAAGTTGGTGG
		β^E	β^E	GGTGAACGTGGATGAAGTTGGTGGT**A**AGGCCCTGGGCAGGTTGGTATCAAG
HbE #2 HSPCs	male	β^{CD17}	β^0	GTCTGCCGTTACTGCCCTGTGGGGC**T**AGGTGAACGTGGATGAAGTTGGTGG
		β^E	β^E	GGTGAACGTGGATGAAGTTGGTGGT**A**AGGCCCTGGGCAGGTTGGTATCAAG
HbE #3 HSPCs	female	β^{CD17}	β^0	GTCTGCCGTTACTGCCCTGTGGGGC**T**AGGTGAACGTGGATGAAGTTGGTGG
		β^E	β^E	GGTGAACGTGGATGAAGTTGGTGGT**A**AGGCCCTGGGCAGGTTGGTATCAAG
IVS II-654 #1 HSPCs	female	$\beta^{CD41/42(-CTTT)}$	β^0	TGGTCTACCCTTGGACCCAGAGGTT**[-CTTT]**GAGTCCTTTGGGGATCTGTCC
		$\beta^{IVS\ II\text{-}654}$	β^+	AACAGTGATAATTTCTGGGTTAAGG**T**AATAGCAATATCTCTGCATATAAAT
IVS II-654 #2 HSPCs	female	$\beta^{CD41/42(-CTTT)}$	β^0	TGGTCTACCCTTGGACCCAGAGGTT**[-CTTT]**GAGTCCTTTGGGGATCTGTCC
		$\beta^{IVS\ II\text{-}654}$	β^+	AACAGTGATAATTTCTGGGTTAAGG**T**AATAGCAATATCTCTGCATATAAAT
IVS II-654 #3 HSPCs	female	β^{-28}	β^+	GGAGGGCAGGAGCCAGGGCTGGGCATA**G**AAGTCAGGGCAGAGCCATCTATT
		$\beta^{IVS\ II\text{-}654}$	β^+	AACAGTGATAATTTCTGGGTTAAGG**T**AATAGCAATATCTCTGCATATAAAT
IVS II-654 #4 HSPCs	female	$\beta^{IVS\ II\text{-}654}$	β^+	AACAGTGATAATTTCTGGGTTAAGG**T**AATAGCAATATCTCTGCATATAAAT
		$\beta^{IVS\ II\text{-}654}$	β^+	AACAGTGATAATTTCTGGGTTAAGG**T**AATAGCAATATCTCTGCATATAAAT

Source Data of Supplementary Fig. 1

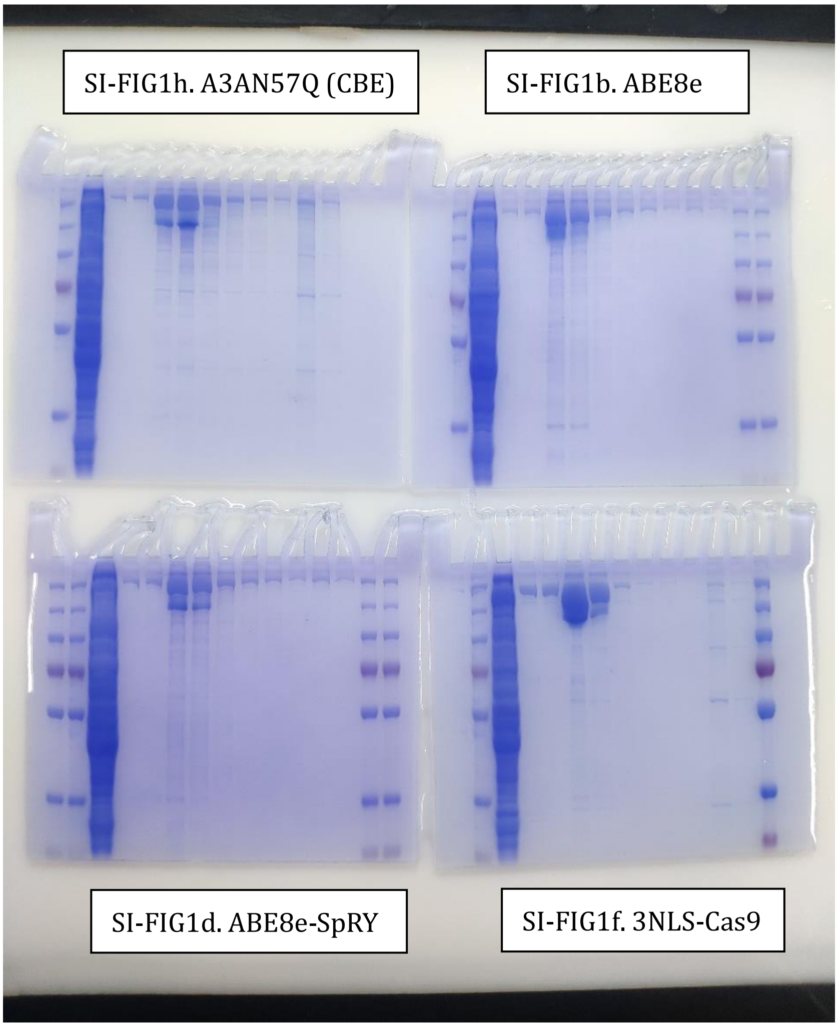

Supplement: Supplementary file 1 — Supplementary Information [file 41467_2022_35508_MOESM1_ESM.pdf]
