# Supplementary material for: Therapeutic adenine base editing of human hematopoietic stem cells
Source: Nat Commun. 2023 Jan 13;14:207. doi: 10.1038/s41467-022-35508-7 (PMC9839747; doi:10.1038/s41467-022-35508-7)
Supplement: Supplementary file 3 — Description of Additional Supplementary Files [file 41467_2022_35508_MOESM3_ESM.pdf]

Description of Additional Supplementary Files

Supplementary Data 1: Single guide RNA sequences, PAM and related primers

Supplementary Data 2: Predicted off-target site information of sg1620 and related primers

Supplementary Data 3: Predicted off-target site information of sgHBGsense and related primers
