# Supplementary material for: Therapeutic adenine base editing of human hematopoietic stem cells
Source: Nat Commun. 2023 Jan 13;14:207. doi: 10.1038/s41467-022-35508-7 (PMC9839747; doi:10.1038/s41467-022-35508-7)
Supplement: Supplementary file 7 — Reporting Summary [file 41467_2022_35508_MOESM7_ESM.pdf]

nature portfolio

Corresponding author(s): Yuxuan Wu, Jiaoyang Liao

Last updated by author(s): Nov 4, 2022

Reporting Summary

Nature Portfolio wishes to improve the reproducibility of the work that we publish. This form provides structure for consistency and transparency in reporting. For further information on Nature Portfolio policies, see our Editorial Policies and the Editorial Policy Checklist.

Statistics

For all statistical analyses, confirm that the following items are present in the figure legend, table legend, main text, or Methods section.

n/a	Confirmed	
☐	☒	The exact sample size (*n*) for each experimental group/condition, given as a discrete number and unit of measurement
☐	☒	A statement on whether measurements were taken from distinct samples or whether the same sample was measured repeatedly
☐	☒	The statistical test(s) used AND whether they are one- or two-sided<br>*Only common tests should be described solely by name; describe more complex techniques in the Methods section.*
☒	☐	A description of all covariates tested
☒	☐	A description of any assumptions or corrections, such as tests of normality and adjustment for multiple comparisons
☐	☒	A full description of the statistical parameters including central tendency (e.g. means) or other basic estimates (e.g. regression coefficient) AND variation (e.g. standard deviation) or associated estimates of uncertainty (e.g. confidence intervals)
☐	☒	For null hypothesis testing, the test statistic (e.g. *F*, *t*, *r*) with confidence intervals, effect sizes, degrees of freedom and *P* value noted<br>*Give P values as exact values whenever suitable.*
☒	☐	For Bayesian analysis, information on the choice of priors and Markov chain Monte Carlo settings
☒	☐	For hierarchical and complex designs, identification of the appropriate level for tests and full reporting of outcomes
☒	☐	Estimates of effect sizes (e.g. Cohen's *d*, Pearson's *r*), indicating how they were calculated

Our web collection on statistics for biologists contains articles on many of the points above.

Software and code

Policy information about availability of computer code

Data collection	BD FACSDiva Software Version 8.0.1
Data analysis	FlowJo V10, CRISPResso2 sotware v2.1.3, GraphPad Prism v9.0.0, FastQC (v0.11.9) and cutadapt (v3.4), STAR (v2.7.9a), GATK (v4.2.1.0), bcftools (v1.13)

For manuscripts utilizing custom algorithms or software that are central to the research but not yet described in published literature, software must be made available to editors and reviewers. We strongly encourage code deposition in a community repository (e.g. GitHub). See the Nature Portfolio guidelines for submitting code & software for further information.

Data

Policy information about availability of data

All manuscripts must include a data availability statement. This statement should provide the following information, where applicable:

- Accession codes, unique identifiers, or web links for publicly available datasets
- A description of any restrictions on data availability
- For clinical datasets or third party data, please ensure that the statement adheres to our policy

All amplicon deep-sequencing data and RNA sequencing data generated in this article can be found at the National Center for Biotechnology Information's Sequence Read Archive with accession code PRJNA892449[https://www.ncbi.nlm.nih.gov/sra/PRJNA892449]. All data supporting the findings of this study are available within the article and Supplementary Information files and also are available from the corresponding author upon reasonable request. Source data are provided with this paper.

Field-specific reporting

Please select the one below that is the best fit for your research. If you are not sure, read the appropriate sections before making your selection.

[x] Life sciences [ ] Behavioural & social sciences [ ] Ecological, evolutionary & environmental sciences

For a reference copy of the document with all sections, see nature.com/documents/nr-reporting-summary-flat.pdf

Life sciences study design

All studies must disclose on these points even when the disclosure is negative.

Sample size	No sample size calculation was performed in this article. The sample sizes for all statistical comparisons were made using the community default criteria, i.e. biological replicates >=3.
Data exclusions	No data were excluded from the analyses.
Replication	Data obtained from edited input cells for transplantation experiments were successfully performed with more than three technical replicates. The cells were then divided equally and transplanted into multiple recipient mice. All the other experiments are replicated more than three times. All attempts at replication were successful.
Randomization	Samples were allocated into experimental groups randomly.
Blinding	The Investigators were not blinded to allocation during experiments and outcome assessment.

Reporting for specific materials, systems and methods

We require information from authors about some types of materials, experimental systems and methods used in many studies. Here, indicate whether each material, system or method listed is relevant to your study. If you are not sure if a list item applies to your research, read the appropriate section before selecting a response.

Materials & experimental systems

n/a	Involved in the study	
[ ]	[x]	Antibodies
[x]	[ ]	Eukaryotic cell lines
[x]	[ ]	Palaeontology and archaeology
[ ]	[x]	Animals and other organisms
[ ]	[x]	Human research participants
[x]	[ ]	Clinical data
[x]	[ ]	Dual use research of concern

Methods

n/a	Involved in the study	
[x]	[ ]	ChIP-seq
[ ]	[x]	Flow cytometry
[x]	[ ]	MRI-based neuroimaging

Antibodies

Antibodies used	Violet 421™ anti-human CD45 Antibody (304032, Biolegend),<br>PE/Dazzle™ 594 anti-mouse CD45 Antibody (103146, Biolegend),<br>PE anti-human CD235a (Glycophorin A) Antibody (349106, Biolegend),<br>FITC anti-human CD33 Antibody (303304, BioLegend),<br>APC anti-human CD19 Antibody (302212, BioLegend),<br>Fixable Viability Dye eFluor 780 for live/dead staining (65-0865-14, Thermo Fisher).
Validation	Each antibody for the species and application is validated. Validation statements are available on the manufacturer's website. Violet 421™ anti-human CD45 Antibody (304032, Biolegend), https://www.biolegend.com/it-it/products/brilliant-violet-421-anti-human-cd45-antibody-7332�PE/Dazzle™ 594 anti-mouse CD45 Antibody (103146, Biolegend), https://www.biolegend.com/fr-fr/products/pe-dazzle-594-anti-mouse-cd45-antibody-10070?GroupID=BLG1932�PE anti-human CD235a (Glycophorin A) Antibody (349106, Biolegend), https://www.biolegend.com/en-gb/products/pe-anti-human-cd235a-glycophorin-a-antibody-6769; FITC anti-human CD33 Antibody (303304, BioLegend), https://www.biolegend.com/en-us/products/fitc-anti-human-cd33-antibody-726; APC anti-human CD19 Antibody (302212, BioLegend), https://www.biolegend.com/ja-jp/products/apc-anti-human-cd19-antibody-715; Fixable Viability Dye eFluor 780 for live/dead staining (65-0865-14, Thermo Fisher), https://www.thermofisher.cn/order/catalog/product/65-0865-14.

Animals and other organisms

Policy information about studies involving animals; ARRIVE guidelines recommended for reporting animal research

Laboratory animals	Female 4-6 weeks NOD.Cg-KitW-41J /ShiLtJGpt-Prkdcem26 Il2rgem26 / Gpt (NCG-X) mice were ordered from GemPharmatech (Nanjing, China) (Stock T003802).
Wild animals	The study did not involve in wild animals.
Field-collected samples	The study did not involve samples collected from the field.
Ethics oversight	All animal experiments were approved by University Committee on Animal Research Protection of East China Normal University.

Note that full information on the approval of the study protocol must also be provided in the manuscript.

Human research participants

Policy information about studies involving human research participants

Population characteristics	Genotype and age-sex information of the cell donors involved in this study are listed below: β-thalassaemia patient (β0β+ #1), 7 yrs old, male, [genotype βCD41/42(-CTTT)/β-28]; HbE #1 patient, 7 yrs old, male, [genotype βCD17/βE]; HbE #2 patient, 8 yrs old, male, [genotype βCD17/βE]; HbE #3 patient, 7 yrs old, female, [genotype βCD17/βE]; IVS II-654 #1 patient, 8 yrs old, female, [genotype βCD41/42(-CTTT)/βIVS II-654]; IVS II-654 #2 patient, 7 yrs old, female, [genotype βCD41/42(-CTTT)/βIVS II-654]; IVS II-654 #3 patient, 8 yrs old, female, [genotype β-28/βIVS II-654]; IVS II-654 #4 patient, 9 yrs old, female, [genotype βIVS II-654/βIVS II-654].
Recruitment	Healthy human participants were recruited anonymously. Thalassaemia patients were recruited based on a doctor's diagnosis of β-thalassemia with genotype confirmation. No potential self-selection bias and other biases are present.
Ethics oversight	Peripheral blood mobilized human CD34+ HSPCs from anonymous healthy donors were obtained from the First Affiliated Hospital of Zhejiang University School of Medicine (FAHZU), approved by the Medical Ethics Committee (MEC) of FAHZU, and informed consent was obtained from the donors. β-thalassemia patient CD34+ HSPCs were isolated from plerixafor-mobilized or unmobilized peripheral blood following Xiangya Hospital Central South University Medical Ethics Committee (MEC), the First Affiliated Hospital of Guangxi Medical University MEC and PLA 923 Hospital MEC approval and informed patient consent.

Note that full information on the approval of the study protocol must also be provided in the manuscript.

Flow Cytometry

Plots

Confirm that:

☒ The axis labels state the marker and fluorochrome used (e.g. CD4-FITC).

☒ The axis scales are clearly visible. Include numbers along axes only for bottom left plot of group (a 'group' is an analysis of identical markers).

☒ All plots are contour plots with outliers or pseudocolor plots.

☒ A numerical value for number of cells or percentage (with statistics) is provided.

Methodology

Sample preparation	Mouse bone marrow were harvested for flow cytometry analysis as described in the manuscript.
Instrument	FACSAria II machine (BD Biosciences)
Software	Flowjo v10
Cell population abundance	hCD45+ for Human chimerism, hCD19 for human B cells, human CD33 for human granulocytes and human monocytes, hCD235a for human erythorid cells
Gating strategy	Human cells gated from hCD45+ population, mouse cells gated from mCD45+ population. B cells gated from hCD45+CD19+ population.Granulocytes gated from hCD45+CD19-CD33dim with SSC high population. Monocytes gated from hCD45+CD19-CD33+ with SSC low population. Erythroid cells gated from hCD45-mCD45-hCD235a+ population.

☒ Tick this box to confirm that a figure exemplifying the gating strategy is provided in the Supplementary Information.
