# Supplementary figures and images for: Therapeutic adenine base editing of human hematopoietic stem cells
Source: Nat Commun. 2023 Jan 13;14:207. doi: 10.1038/s41467-022-35508-7 (PMC9839747; doi:10.1038/s41467-022-35508-7)

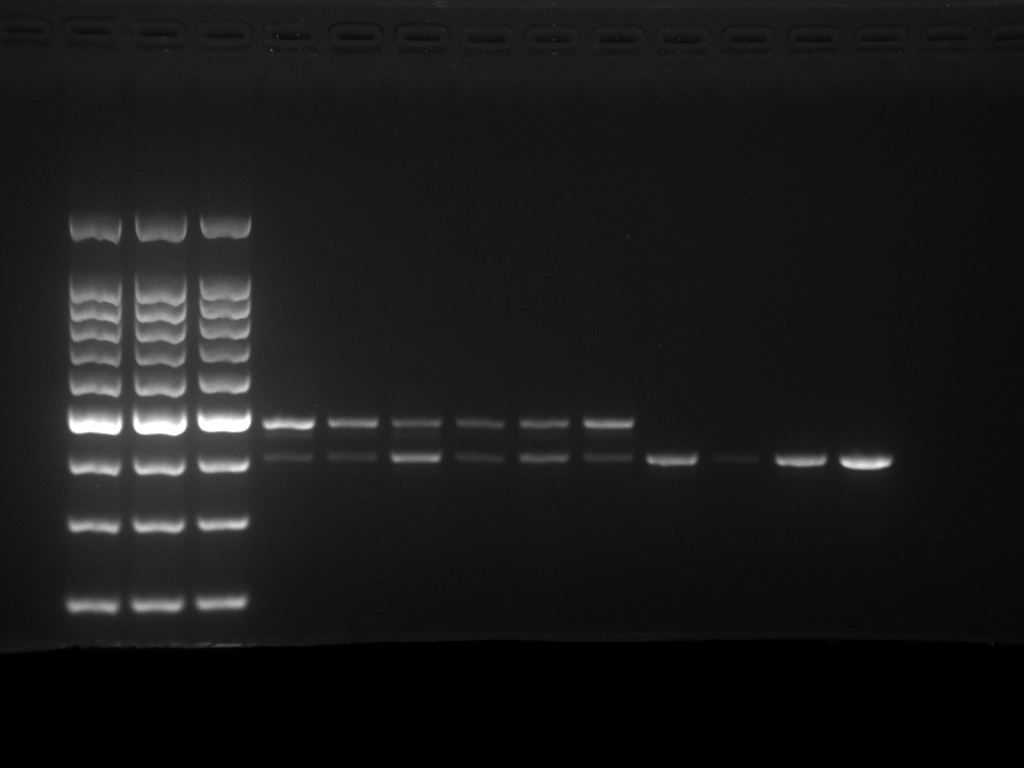

Supplement: Supplementary file 8 — Source Data [file 41467_2022_35508_MOESM8_ESM.zip › Source Data/Source Data Figure4f-1.Tif]

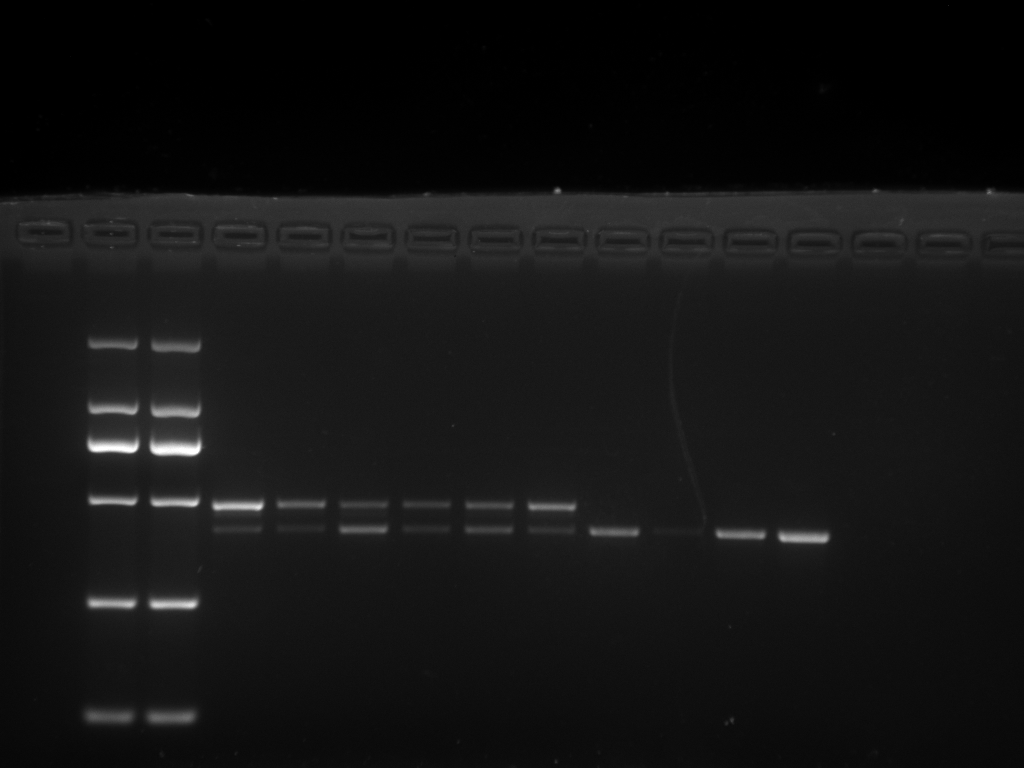

Supplement: Supplementary file 8 — Source Data [file 41467_2022_35508_MOESM8_ESM.zip › Source Data/Source Data Figure4f-2.Tif]

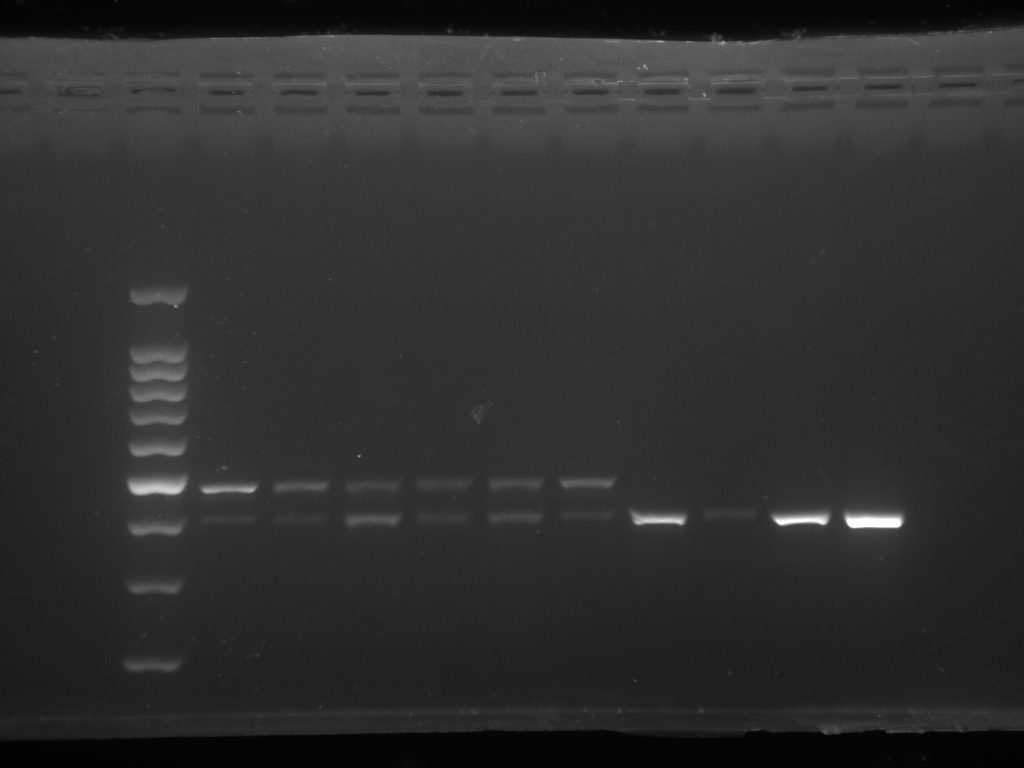

Supplement: Supplementary file 8 — Source Data [file 41467_2022_35508_MOESM8_ESM.zip › Source Data/Source Data Figure4f-3.Tif]
